# The Potential of Artificial Intelligence to Detect Lymphovascular Invasion in Testicular Cancer

**DOI:** 10.3390/cancers13061325

**Published:** 2021-03-16

**Authors:** Abhisek Ghosh, Korsuk Sirinukunwattana, Nasullah Khalid Alham, Lisa Browning, Richard Colling, Andrew Protheroe, Emily Protheroe, Stephanie Jones, Alan Aberdeen, Jens Rittscher, Clare Verrill

**Affiliations:** 1 Department of Cellular Pathology, Oxford University Hospitals NHS Foundation Trust, John Radcliffe Hospital, Oxford OX3 9DU, UK; Lisa.Browning@ouh.nhs.uk (L.B.); Richard.colling@ouh.nhs.uk (R.C.); Clare.Verrill@ouh.nhs.uk (C.V.); 2 Nuffield Department of Clinical and Laboratory Sciences, Oxford University, John Radcliffe Hospital, Oxford OX3 9DU, UK; 3 Big Data Institute, Li Ka Shing Centre for Health Information and Discovery, University of Oxford, Oxford OX3 7LF, UK; korsuk.sirinukunwattana@eng.ox.ac.uk (K.S.); nasullah.khalidalham@eng.ox.ac.uk (N.K.A.); jens.rittscher@eng.ox.ac.uk (J.R.); 4 Oxford NIHR Biomedical Research Centre, Oxford University, Oxford OX3 9DU, UK; 5 Institute of Biomedical Engineering, Department of Engineering Science, University of Oxford, Oxford OX3 7DQ, UK; 6 Ground Truth Labs, Oxford OX4 2HN, UK; alan@groundtruthlabs.com; 7 Nuffield Department of Surgical Sciences, Oxford University, Oxford OX3 9DU, UK; stephanie.jones@nds.ox.ac.uk; 8 Department of Oncology, Oxford University Hospitals NHS Foundation Trust, John Radcliffe Hospital, Oxford OX3 9DU, UK; andrew.protheroe@oncology.ox.ac.uk (A.P.); EXP819@student.bham.ac.uk (E.P.)

**Keywords:** testicular cancer, germ cell tumours, lymphovascular invasion, deep learning, artificial intelligence

## Abstract

**Simple Summary:**

Testicular cancer predominantly affects young adult men and is the most common cancer affecting this demographic. An important prognostic factor for early-stage disease is the presence of tumours within blood vessels or lymphatic channels, which is termed lymphovascular invasion. This is identified by careful microscopic examination of the tumour after orchidectomy, which is frequently challenging and time-consuming. We trained a proof-of-concept deep learning artificial intelligence algorithm to automatically identify areas suspicious for lymphovascular invasion in digital whole slide images from testicular tumours. Our study demonstrates that automated detection of areas suspicious for lymphovascular invasion by artificial intelligence algorithms is feasible and may prove useful in the context of a decision support tool.

**Abstract:**

Testicular cancer is the most common cancer in men aged from 15 to 34 years. Lymphovascular invasion refers to the presence of tumours within endothelial-lined lymphatic or vascular channels, and has been shown to have prognostic significance in testicular germ cell tumours. In non-seminomatous tumours, lymphovascular invasion is the most powerful prognostic factor for stage 1 disease. For the pathologist, searching multiple slides for lymphovascular invasion can be highly time-consuming. The aim of this retrospective study was to develop and assess an artificial intelligence algorithm that can identify areas suspicious for lymphovascular invasion in histological digital whole slide images. Areas of possible lymphovascular invasion were annotated in a total of 184 whole slide images of haematoxylin and eosin (H&E) stained tissue from 19 patients with testicular germ cell tumours, including a mixture of seminoma and non-seminomatous cases. Following consensus review by specialist uropathologists, we trained a deep learning classifier for automatic segmentation of areas suspicious for lymphovascular invasion. The classifier identified 34 areas within a validation set of 118 whole slide images from 10 patients, each of which was reviewed by three expert pathologists to form a majority consensus. The precision was 0.68 for areas which were considered to be appropriate to flag, and 0.56 for areas considered to be definite lymphovascular invasion. An artificial intelligence tool which highlights areas of possible lymphovascular invasion to reporting pathologists, who then make a final judgement on its presence or absence, has been demonstrated as feasible in this proof-of-concept study. Further development is required before clinical deployment.

## 1. Introduction

Testicular cancer is the most common cancer in men under 45, with the vast majority being testicular germ cell tumours (TGCT). With modern therapeutic regimes, these tumours have an extremely high cure rate greater than 90% overall, but challenges still remain [1]. Current stratification tools are imperfect, resulting in both under and over-treatment. Some groups of patients do poorly, and 20–30% show resistance to standard chemotherapeutic agents, with extremely limited subsequent therapeutic options [2]. Four hundred men per year die of TGCT in the United States (US) at a median age of 30 [3].

Patients are usually treated with primary orchidectomy, and the tumour type is ascertained histologically using the World Health Organisation (WHO) classification system [4], where tumours are broadly divided into those that are derived from the precursor lesion Germ Cell Neoplasia In-Situ (GCNIS) or not. Within the GCNIS derived lesions, tumours can be divided broadly into seminoma or non-seminomatous germ cell tumours (NSGCT), with the latter generally being more aggressive. TGCT are notoriously heterogenous as they can be mixed germ cell tumours composed in any combination of the elements of seminoma, embryonal carcinoma, yolk sac tumour (post pubertal type), teratoma (post pubertal type) and choriocarcinoma.

The generally good prognosis of these tumours makes powering of studies to evaluate prognostic factors difficult, with much evidence based on large cohort studies. One of the few parameters that is a powerful predictor for metastasis or disease recurrence in stage I disease [5] is the presence of lymphovascular invasion (LVI) in NSGCT [6]. The evidence is summarised in several review articles, and the risk of an adverse outcome varies in the literature from approximately 46–62% in NSGCT when LVI is present [7]. The evidence for LVI is less clear in seminoma, with some studies demonstrating an adverse impact on outcome [8] and others not [9,10]. Other pathological features associated with adverse prognosis include tumour size [11,12], invasion of structures, such as the hilum [13] and rete testis stroma [9], although the evidence remains less strong than for LVI. The presence of embryonal carcinoma or predominance of this component within a tumour (for example, comprising >50% of the tumour) [14] has a similar predictive power for metastasis on a meta-analysis [15], but there is no agreed-upon way to assess for its percentage.

TGCT are often managed in supra-regional networks. For example, in the United Kingdom (UK), these cover a population of 2–4 million and manage 50–100 new patients per year. This means that expertise, including pathological expertise, is concentrated in specialist centres. Specialist assessment for LVI is valuable, as identification of genuine LVI is often challenging. Tumour may be artefactually displaced into vessels during specimen cut up or processing. Atypical histiocytes within vessels, intratubular tumour and retraction artefact, may also be mistaken for LVI [16,17,18]. Central pathology review of TGCTs aims to improve the reproducibility of factors such as LVI assessment. This approach is supported by limited evidence; one study showed 27% of cases reviewed at a central pathology laboratory were reclassified as containing LVI, and 19% were reclassified as containing no LVI; only the centrally reviewed LVI assessment correlated with node metastases [19].

LVI can be present within the tumour, spermatic cord, tunica albuginea or hilar soft tissue. Regardless of location, its presence is regarded as TNM category pT2 [18,19]. As the presence of LVI may trigger adjuvant chemotherapy, accurate assessment of this parameter is vital, and when its presence is uncertain, it is recommended that it is considered equivocal and assigned as ‘not identified’ (no LVI is present) to avoid triggering unnecessary chemotherapy [6,7,16].

Assessment for LVI by pathologists is inherently limited by being undertaken by human observers. Examination of large areas of tumour for LVI is time-consuming and challenging, as foci of LVI may be small and subjective. Nonetheless, the presence of LVI may markedly affect patient management when present, and identification of a single focus deemed to be genuine is enough to mark a case as positive. As such, automated identification of foci likely to represent LVI may be of significant clinical utility.

Digital pathology (DP) refers to the generation of whole slide images from histology slides, which can be viewed on a screen to form a diagnostic report. Histological diagnosis and pathological staging by cellular pathologists have traditionally been achieved using glass slides and microscopy [20,21]. There is now a significant push for implementation in laboratories of DP, and digitally-enabled care is seen as a core component of health service planning to increase efficiency, network working and improve quality [22,23]. In the UK, the Government’s Industrial Life Sciences Strategy highlighted pathology as being “ripe” for innovation by the use of DP and artificial intelligence (AI) [24]. There is great potential for the use of AI to assist pathologists and derive novel biological insights into disease biology, which are not appreciable by human observers [25]. As many pathology departments do not have sufficient pathologists for the workload, it is important to explore the potential of these technologies [26].

AI algorithms utilising convolutional neural networks (CNNs) for image analysis have already shown significant promise in the pathological assessment of a range of tumours, including screening for prostate cancer in prostate biopsies [27,28], providing novel assessments of clinical outcome [29,30] or predicting the presence of mutations [31] or molecular subtypes [32] from haematoxylin and eosin (H&E) stained sections. The utility of such algorithms in the identification of small areas of prognostic significance in digital whole slide images has been demonstrated previously in the context of identifying metastatic breast cancer within lymph nodes [33,34].

There is relatively sparse literature on the use of AI in TGCT, due to the challenges of training and validating algorithms in these heterogeneous tumours, as well as the relative rarity of these tumours and their concentration in specialist centres. One AI study evaluated the ability of a deep learning model to assess tumour infiltrating lymphocytes (TILs) in both seminoma and NSGCT. An AI algorithm was able to evaluate lymphocyte density in tumours beyond the capacity of human visual assessment, counting more than 100,000 cells per sample. Although previous studies involving human observers had failed to identify lymphocyte density as a significant prognostic factor, the AI tool was able to use lymphocyte density to predict clinical stage and disease relapse in seminoma [35].

In this study, we demonstrate a proof-of-concept AI algorithm that aims to highlight foci of likely LVI for the attention of the reviewing pathologist, who then ultimately makes the decision as to the presence or absence of LVI.

## 2. Materials and Methods

### 2.1. Patients

This study was conducted under the Oxford Radcliffe Biobank (ORB) Research Ethics Approval (reference 19/SC/0173). A total of 29 cases of primary TGCT were retrospectively selected and included in sequence from the period January 2019 to July 2020 from the Cellular Pathology Department archives of the John Radcliffe Hospital, Oxford, after a check of the research consent section of the procedure consent form. These were cases that had primary management within the trust (i.e., had orchidectomy in Oxford, and not referrals). This reflects the typical number of cases seen of this tumour type in the department. Sixteen of the tumours were pure seminomas, and 13 were NSGCTs (including 10 mixed germ cell tumours and 1 spermatocytic tumour). One prepubertal type teratoma was also included to increase the cohort size, acknowledging that it is not a malignant TGCT. Ten patients had metastatic disease at presentation, and one patient later developed metastatic disease. Thirteen of the patients went on to have adjuvant chemotherapy.

### 2.2. Digitisation of Slides

Three hundred and two archived whole slide images from these patients were exported in TIFF format to the Visiopharm platform, using the Philips De-ID tool (Version 1.1.5, Philips Digital Pathology Solutions Document DP-174226) with all personally identifiable information removed. Images were imported into our in-house annotation platform, Annotation of Image Data by Assignments (AIDA) [32,36]. Only slides showing sections sampled from testicular parenchyma were included. It is acknowledged that LVI can be seen in other blocks, such as cord blocks, but due to the infrequent nature, we did not focus on those in this study.

Slides were derived from 4–5 µm thick sections cut from formalin-fixed, paraffin-embedded blocks of tissue, stained with H&E. Slides were scanned using the Philips IntelliSite Ultrafast Scanner using a 40× objective.

### 2.3. Training the Model

Information from the deidentified reports was used to split the patient cohort (Table 1) into a training set of 19 cases (comprising 184 whole slide images) and a validation set of 10 cases (comprising 118 whole slide images). Six of the training set cases and 3 of the validation set cases were reported to have confirmed LVI (32% and 30%, respectively). The training set included 11 cases of pure seminoma and 8 cases of NSGCT. This set included the 1 prepubertal teratoma and 1 spermatocytic tumour, as well as 6 mixed germ cell tumours. The validation set included 5 cases of pure seminoma and 5 cases of NSGCT (4 of which were mixed germ cell tumours).

Three hundred and fifty candidate foci were manually annotated by a pathologist (AG) on 141 of the 184 training whole slide images using the Visiopharm platform. Annotated slides were then exported to the AIDA platform. Each candidate focus was then reviewed by two specialist uropathologists (CV, RC) and classified as to whether LVI was considered present, equivocal or not present as per the International Collaboration on Cancer Reporting (ICCR) classification [6]. Equivocal foci were defined as those that would be appropriate to flag to the attention of a pathologist but not considered genuine LVI. One hundred and fifty-four foci in which LVI was considered not present by both reviewers were removed as labels on Visiopharm.

The Visiopharm AI module uses manually classified annotations to train a CNN for automatic segmentation of image structures. The Deeplabv3+ semantic segmentation architecture was used [37]. This neural network extracts features from input images through multiple layers of processing, aggregating features at multiple scales. Training using manual annotations teaches extraction of appropriate features and generates a model which is able to segment images into areas according to the input classifications.

Following initial training, the model was applied to the remaining 43 training slides, and parameters were empirically adjusted to select for areas with a high prediction confidence for LVI only. These areas were initially reviewed by another pathologist (AG) to remove any clearly misclassified areas and add additional candidate foci to produce 121 more foci for review. These foci were then reviewed by three specialist uropathologists (CV, RC, LB) and again classified depending on whether LVI was considered present, equivocal or not present. The uropathologists used for final classification have 13 years (CV), 2 years (RC) and 11 years (LB) experience post-specialist registration and regularly review cases for the supraregional germ cell MDT. CV is the supraregional pathology lead for the Thames Valley germ cell tumour network.

**Figure 1:**
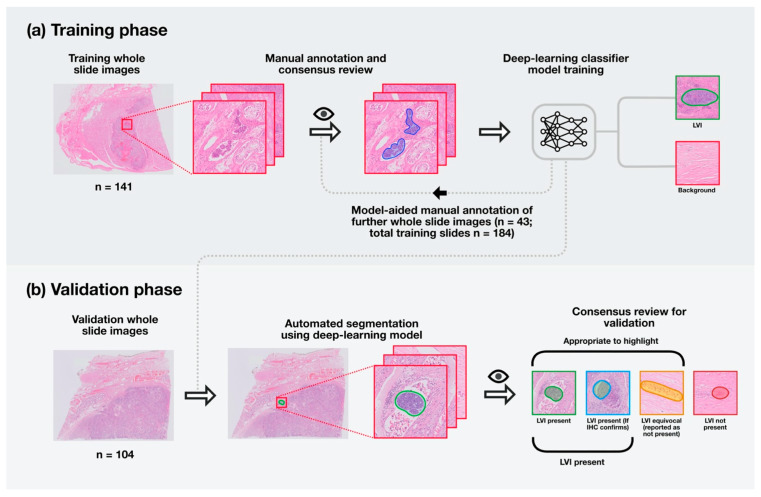
Summary of training and testing of a deep-learning segmentation model for identifying regions of lymphovascular invasion (LVI) in testicular cancer. (**a**) One hundred and forty-one digitised whole slide images were annotated manually, and consensus review by expert pathologists was performed to determine foci appropriate to use for training. These foci were used to train a deep-learning classifier to segment areas with a high prediction probability for LVI. The trained model was applied to a further 43 digitised whole slide images, which were again manually reviewed by specialist uropathologists, and the resulting annotations were used to tune the classifier. The total training set included 184 whole slide images from 19 patients. (**b**) One hundred and four digitised whole slide images from nine distinct cases were used for final validation. Each image was processed through the classifier, and the detected foci were reviewed independently by three specialist pathologists. A majority vote was used to determine ground truth as to areas appropriate to highlight and areas with LVI present.

Eighteen foci that were considered not appropriate to flag by all reviewers were then removed as labels on Visiopharm, and a further round of training performed on the entire labelled training set. The final model was configured to highlight pixels with high prediction confidence only and predicted foci dilated to combine those in close proximity to each other and aid subsequent human review. An overview of the training process is shown in Figure 1a.

### 2.4. Assessment of Model Performance

The model algorithm was applied to the validation set of 118 whole slide images. A quality check was performed on the image and the detected foci to exclude tissue processing artefacts. One NSGCT case, encompassing 14 whole slide images, was excluded due to failure of the algorithm within a stroma-rich tumour, a morphology that was not represented in the training set (See results). A final set of 104 whole slide images was used for testing algorithm performance.

Thirty-four foci within 12 slides were identified by the algorithm for evaluation. Each of these foci was then evaluated by the three uropathologists using the AIDA platform. The pathologists were instructed to classify each focus depending on whether LVI was considered present (including cases where confirmatory immunohistochemistry (IHC) would be used), equivocal or not present. An overview of the validation process is shown in Figure 1b.

Each focus was categorised based on the majority vote from the three reviewers. If a focus was considered to contain LVI or was considered equivocal for LVI by two or more of the three reviewers, the focus was considered appropriate to flag. If a focus was considered to contain LVI by two or more of the three reviewers, the focus was considered to contain LVI by consensus. To reflect the real-world usage of the tool, the overall precision was calculated, defined as the number of appropriate foci identified divided by the total number of foci identified. Precision was also calculated separately for consensus LVI.

The detected foci were ordered by area on the basis that multiple adjacent high probability foci may have been combined during post-processing, and these areas should represent the first foci to review, as these are the areas most likely to be true positives. Precision-recall curves were used to assess performance on the ordered results. To estimate sensitivity (recall) in the validation set for the construction of a precision-recall curve, a total of 124 more candidate foci, in addition to those identified by the algorithm, were annotated on the validation slides by another pathologist (AG). These foci were independently reviewed by an expert uropathologist (CV) and classified depending on whether LVI was considered present, equivocal or not present. Estimated recall for appropriate areas was defined as the number of appropriate areas detected, divided by this figure plus the total number of additional appropriate foci identified on review by the single expert pathologist. Similarly, estimated recall for LVI was defined as the number of areas of consensus LVI detected, divided by this figure plus the total number of additional definite LVI foci identified on review by a single expert pathologist.

### 2.5. Statistical Analysis

Interobserver variability was estimated by Fleiss kappa statistics, performed on the classification of each focus by the three independent uropathologists. Kappa statistics were calculated separately based on the classification of foci as LVI or not, as well as the classification of foci as appropriate to flag or not. Interpretation of the kappa statistic was based on previously published thresholds, suggesting; <0 as poor or no agreement, 0–0.2 as slight agreement, 0.21–0.40 as fair agreement, 0.41–0.60 as moderate agreement, 0.61–0.80 as substantial agreement, and 0.81–1.00 as almost perfect agreement [38].

For the purposes of statistical evaluation, no distinction was made between foci that were classified as definite LVI and those that a pathologist would classify as definite LVI with the aid of immunohistochemistry.

The number of foci of consensus LVI was compared between cases with metastatic disease at presentation (or who developed metastatic disease subsequently) and those without metastatic disease using the unpaired *t*-test.

## 3. Results

### 3.1. Classifier Precision

**Figure 2:**
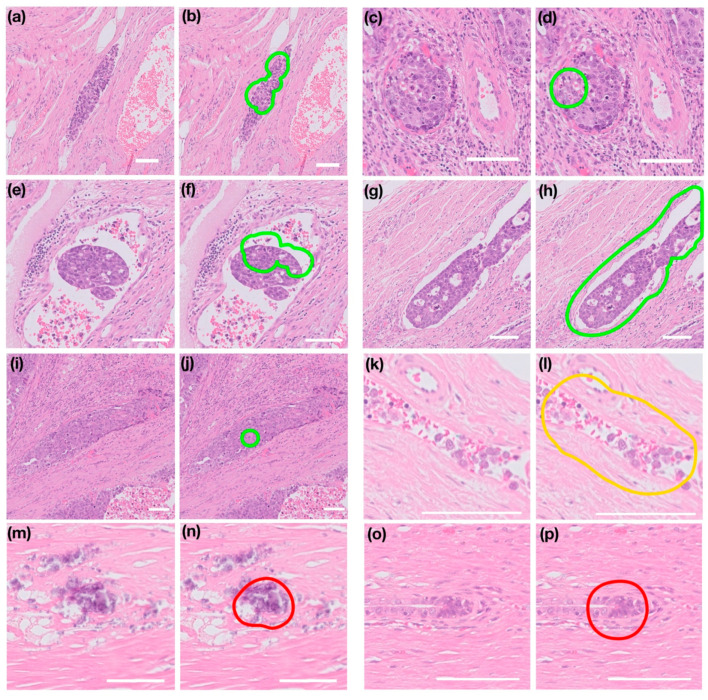
Examples of foci flagged by the deep-learning classifier model. Original images are shown in (**a**,**c**,**e**,**g**,**i**,**k**,**m**,**o**) and classifier output in (**b**,**d**,**f**,**h**,**j**,**l**,**n**,**p**). Images (**a**–**j**) show foci in which the presence of lymphovascular invasion (LVI) was agreed by consensus. Images (**k**,**l**) show a misclassified focus which was considered appropriate to highlight but negative for LVI (i.e., ‘equivocal’). Images (**m**–**p**) show examples of misclassified foci, (**m**,**n**) are embryonal carcinoma in tunica albuginea, and (**o**,**p**) are rete epithelium. Images are shown at varying magnification (scale bar is 100 µm in each).

**Figure 3:**
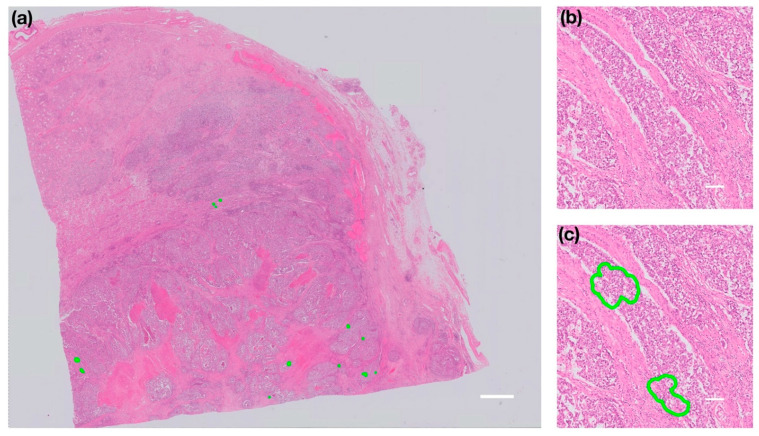
Examples of foci misclassified as LVI within the one failed case. Image (**a**) shows a contrast-enhanced example whole slide image with detected foci highlighted in green. Image (**b**) shows an example high power view of this stroma-rich tumour, and image (**c**) highlights the misclassified foci, in which non-neoplastic stroma mimics blood vessel walls. (Scale bar is 3 mm in the whole slide image and 100 µm in the magnified areas).

The deep-learning classifier identified 34 foci across 104 validation whole slide images. Examples of the detected areas are presented in Figure 2. An example slide and focus from the case in which the algorithm failed is shown in Figure 3. In this stroma-rich tumour, tumour was extensively present adjacent to the non-neoplastic stroma, mimicking tumour-containing blood vessels. This tumour morphology was not included in the training dataset and was, therefore, excluded from the final validation review.

**Figure 4:**
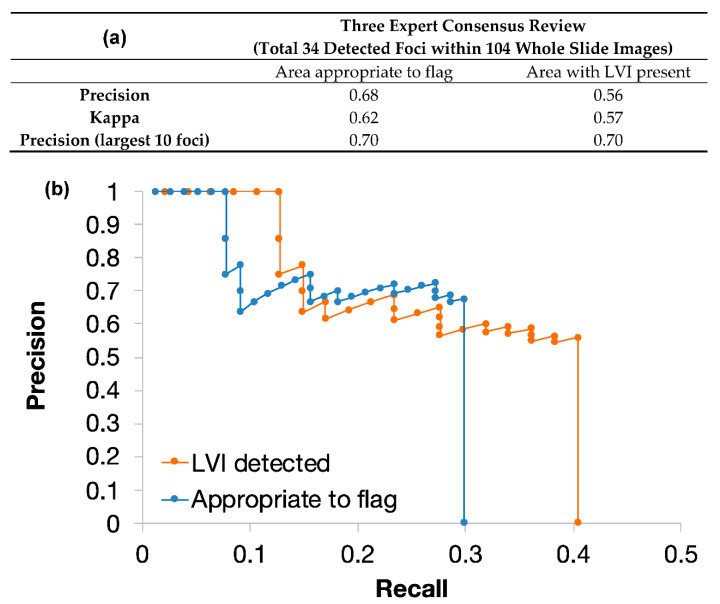
(**a**) Overall precision of the deep-learning classifier following three expert reviews of each identified focus and agreement between experts when classifying into areas appropriate for review or areas of definite LVI. (**b**) Precision-recall curves showing performance of the deep-learning classifier ordered by focus size (a larger focus size indicating a higher number of adjacent high probability pixels).

Twenty-nine of the 34 identified foci were peritumoural, and 5 were intratumoural. Twenty-three of the 34 identified foci were assessed as being appropriate to flag based on consensus expert review, comprising foci that were categorised as LVI (including those that would be confirmed using immunohistochemistry), and those which were considered equivocal (but ultimately negative) [6,7,16]. The overall precision in identifying areas appropriate to flag was 0.68 (Figure 4). Of the areas identified appropriate to flag, 19 contained embryonal carcinoma, 2 contained seminoma, and 1 contained yolk sac tumour.

Nineteen of the 34 identified foci were assessed as containing LVI (including those that would be confirmed using immunohistochemistry), and all of these contained embryonal carcinoma. Of the 15 foci not considered LVI, 5 contained smear artefact (examples in Figure 2k,l), 2 contained lymphoid cells within connective tissue, 2 contained hyperchromatic areas of rete epithelium (examples in Figure 2m,n), 2 contained congested background blood vessels, 1 contained embryonal carcinoma within tunica albuginea (example in Figure 2o,p) and 1 contained a focus of embryonal carcinoma within the stroma. Whilst smear artefact is not genuine LVI, such areas would often require expert consideration depending on the extent, and it was considered valuable to bring such areas to the attention of a reviewing pathologist.

The overall precision of the classifier in identifying areas containing consensus definite LVI (or LVI to be confirmed with immunohistochemistry) was 0.56 (Figure 4).

### 3.2. Interobserver Variability

The kappa statistic for interobserver agreement between three expert pathologists based on the categorisation of each focus as appropriate to flag or not was 0.62 (substantial agreement). Unanimous agreement for appropriateness to flag was seen in 25 foci (74%).

The kappa statistic for interobserver agreement between three expert pathologists based on the categorisation of each focus as LVI or not was 0.57 (moderate agreement). Unanimous agreement for the presence or absence of LVI was seen in 23 foci (66%).

### 3.3. Ranked Retrieval Results

The 34 foci identified by the deep-learning classifier were ranked based on focus size. The top five foci were all classified as appropriate to flag, with LVI deemed to be present in each on consensus review. The overall precision of the top 10 foci for categorisation as appropriate to flag or not was 0.7, and all of the appropriate foci identified in this top 10 were deemed to be LVI on consensus review. This is summarised in Figure 4.

### 3.4. Metastatic Disease

There was no evidence of a significant association between the number of LVI foci identified and the incidence of metastatic disease at presentation in this study (*p* = 0.96).

## 4. Discussion

Examination of H&E-stained histological slides of TGCTs is an important part of the clinical decision-making process in these cancers, and the presence or absence of LVI is a powerful predictor for relapse or metastatic disease [6]. To our knowledge, there are no other examples of the utilisation of artificial intelligence techniques to detect lymphatic or vascular invasion in cancer histology. Identification of LVI often informs the decision to administer chemotherapy to patients with stage 1 disease.

In this study, we demonstrated a proof-of-concept deep learning based-approach to identifying candidate foci of LVI within digitised whole slide images of H&E stained sections.

Deep learning is a machine learning technique in which artificial neural networks are instructed to learn from large amounts of training data and progressively improve performance at a specific task. CNNs are a class of such neural networks, which have shown great promise in a range of medical applications, including diagnostic support for pathologists.

Deep learning techniques are often applied to situations where a ground truth measurement is clearly evident. However, morphological diagnosis of LVI is one of a range of problems where significant interobserver variability exists [16,17]. The identification of tumour within a vessel is only part of the difficulty; tumour is frequently artefactually displaced into vessels, and as such, pathologists must consider a range of less easily definable contextual features to come to a decision about whether genuine LVI is present. Immunohistochemistry may sometimes be used in challenging cases, but it is of limited value and is not recommended for routine use [6,7,16].

As well as subjectivity in its diagnosis, the challenge of LVI identification is often compounded by only the focal presence within sections. As such, it is an unbalanced task, and the evaluation by automated tools for its identification is different compared to problems of tumour categorisation.

We have demonstrated in this study that a deep learning classifier is able to identify small areas within whole slide images with a high probability of being genuine LVI. Only one focus of genuine LVI is required to mark a case as positive and hence potentially trigger subsequent management interventions. Indeed, in this study, there was no evidence of a statistically significant association between the number of LVI foci and metastatic disease at presentation, although this cohort may be too small to draw a definite conclusion, and further work is required.

As such, an automated tool with a relatively high precision may lead to significantly increased efficiency when assessing large areas of tissue. In this study, we showed that the model was able to identify foci of LVI at a precision of 0.56, which increased to 0.68 if including foci that required expert human consideration, but were eventually considered equivocal (and thus negative [6,7,16]). The latter figure of 0.68 is the most important as the tool is designed to highlight to pathologists when LVI might be present, not make the decision of when it is present. These precision values should be interpreted in the context of the inherent subjectivity in the interpretation of LVI and/or which foci pathologists would deem appropriate to flag, and thus the ground truth is also inherently subjective; indeed, unanimous agreement for the presence or absence of LVI amongst three pathologists was seen in only 66% of the flagged foci. However, this would be considered as an acceptable or moderate level of agreement for pathologist-based agreement [39]. Our results suggest that only a small number of flagged areas would need to be examined to generate a high probability of finding a consensus positive focus.

Furthermore, it is possible to rank retrieval using a variety of parameters. In this study, we have ranked each focus by size, based on the rationale that many very high probability pixels in close proximity are more likely to represent a human-appreciable area of genuine LVI. When evaluating the highest-ranked foci in this way, the largest five foci were all classified as LVI on consensus review. Other methods of ranking foci could include the distance from the main tumour mass, as foci of tumour in vessels distant from the mass may be more likely to be interpreted as genuine LVI. Further work is required to investigate automated ranking in this way. Rational ranking of identified foci is likely to increase the precision of such a tool in practice greatly.

The challenging nature of agreeing on genuine LVI was demonstrated by measuring interobserver variability in the validation slides. Moderate agreement was reached when deciding whether LVI was present or not (κ = 0.57; Figure 4), which is similar to the slide-level rate of interobserver variability seen as part of a previous study in NSGCTs [18]. The discrepancy between experts can be seen for a variety of reasons, but ultimately, it is an opinion-based judgement and would be subject to variables such as experience, level of fatigue and individual differences as to when a focus has met the threshold for genuine LVI. There are attempts to minimize these by producing international guidelines and strict criteria for which features constitute genuine LVI [16], but there still remain individual differences in interpretation of parameters such as LVI versus mimics, such as smear artefact or intratubular carcinoma [17,18]. In diagnostic practice, these cases would often require assessment and agreement by two or more pathologists. Our majority vote approach to the ground truth replicates this approach. Other similar scenarios, such as the agreement between pathologists as to the presence or absence of extraprostatic extension in prostatectomy specimens, show similar levels of discrepancy [40,41]. The agreement level highlights the difficulty in training and assessing algorithms based on subjective morphological features but emphasises the value of a tool to identify candidate regions, with the pathologist ultimately making the decision.

Our study included a training set of 184 whole slide images from 19 patients and a final validation set that included 104 whole slide images from 9 patients. This reflects the annual number of cases of this relatively uncommon tumour type originating in our supraregional centre; datasets are inherently small in this tumour type, and the availability of high-quality, curated datasets is limited. Although the relatively small number of cases is a limitation, germ cell tumours are highly heterogeneous, creating a diverse training and validation set from these cases, with 10 of the cases representing mixed germ cell tumours. Furthermore, as our approach used data sampled across whole slide images, rather than a more selective patch-based approach, a large amount of training and validation data was available. The number of whole slide images in our dataset is comparable to those in previous studies investigating other histopathological features of prognostic importance [34,35,42]. Future development of the tool would leverage publically available datasets and national/international networks and collaborators to address this.

Further training may help to exclude misclassified areas that are more readily recognised by pathologists as negative. The failure of the algorithm in one NSGCT case likely reflects this morphology not being present in the training set, which is a problem associated with the great morphological heterogeneity of TGCTs; additional training may also help increase the reliability of the tool. In a previous study evaluating TILs by AI in TGCT, the algorithm failed in 31.8% NSGCT and 14.5% seminomas [35]. Many of the detected areas represented foci of embryonal carcinoma (Figure 2), and future studies involving larger numbers of different tumour types within vessels are required to evaluate the tool further and assess sensitivity. Although the evidence for LVI as a prognostic factor in seminoma is less clear [8,9,10], the loosely cohesive nature of such tumours increases the chance of smear artefact [17]. More cases of seminomatous LVI could be included in future studies.

Our approach in this study was to focus on high specificity, i.e., areas with a high probability of being considered as LVI. An alternative approach would be to focus on sensitivity, but the flagging of large numbers of foci at lower specificity diminishes the value of the tool, as this is little different from primary screening for LVI by a pathologist. This does, however, raise an important training point for pathologists; those using such tools should understand the functionality and appreciate that even if a case is not flagged as having LVI, it may still be present, and a full screen of the case still needs to be undertaken. The ’roadmap’ to taking proof-of-concept tools such as this on through to full diagnostic practice is a complex one [43]. We do not claim that this tool makes the assessment of definitive LVI, but that it highlights to pathologists when a case is likely to contain LVI such that the pathologist can assess those areas first, potentially saving time and reducing the risk of missing such areas in some cases. We acknowledge that this tool would require further versions with more training and validation, including cases from multiple centres and validation by multiple expert pathologists from different centres before being tested in a real-life laboratory setting.

AI can be used to support pathologists as described in this study, but it can also be used to derive novel biological insights, not possible with a human observer. Although a key focus in other tumour types, to our knowledge, no studies exist predicting molecular changes in TGCT from morphological appearances (morpho-molecular correlation), and there are no molecular tests that currently guide clinical practice as in other tumour types, which would make identification of mutations of high importance. These tumours show a low rate of mutations compared to common cancers, which would make the prediction of mutations by AI more challenging [44,45], although somatic mutations of the KIT gene and its downstream mediators encoded by the KRAS and NRAS genes have shown significance in seminoma [45]. The primary somatic feature in the development of these tumours is highly recurrent chromosome arm level amplification and reciprocal deletions [46], with copy number gain of chromosome 12p being almost universal in TGCT [46,47]. As novel biomarkers emerge [48], morpho-molecular correlation aided by AI may prove a helpful adjunct to determine the optimal therapeutic approach.

## 5. Conclusions

We have shown in this study that deep learning algorithms have the potential to detect features including LVI, which are considered subjective even by human pathologists. In addition to potential workflow and efficiency benefits in the context of a fully digitised system, such algorithms may prove useful as decision support tools to improve diagnostic reliability.

## Figures and Tables

**Table 1:** Summary of cohort and total numbers of foci of possible lymphovascular invasion (LVI) used for training. One non-seminomatous germ cell tumour (NSGCT) case (14 whole slide images) was excluded from validation assessment following a quality check (see results).

Cohort Summary	Training Set	Validation Set
Cases	19	10
Seminoma	11	5
Non-seminoma	8	5
Whole slide images	184	118
Round 1	141	-
Round 2	43	
Total initially annotated LVI Candidate foci	471	-
Round 1	350	
Round 2	121	
Total foci used for training (after consensus review)	272	-
Round 1	196	
Round 2	76	

## Data Availability

The data presented in this study are available on request from the corresponding author. Images and annotations may be available on request by separate arrangement with Oxford University Innovation via approach to the corresponding author.

## Abbreviations

AI	Artificial intelligence
AIDA	Annotation of Image Data by Assignments
CNN	Convolutional neural networks
DP	Digital pathology
GCNIS	Germ cell neoplasia in-situ
H&E	Haematoxylin and eosin
IHC	Immunohistochemistry
ICCR	International Collaboration on Cancer Reporting
LVI	Lymphovascular invasion
NSGCT	Non-seminomatous germ cell tumour
ORB	Oxford Radcliffe Biobank
TGCT	Testicular germ cell tumour
TIL	Tumour infiltrating lymphocyte
WHO	World Health Organisation
